# Problem-oriented or solution-oriented? The effects of climate narratives on pro-environmental behavioral intention

**DOI:** 10.3389/fpsyg.2026.1878805

**Published:** 2026-08-24

**Authors:** Yuqiang Zhang, Lin Dong

**Affiliations:** 1 School of Government, Yunnan University, Kunming, China; 2 School of Humanities and Law, Northeastern University, Shenyang, China

**Keywords:** climate communication, narrative, narrator, pro-environmental behavioral intention, environmental self-efficacy

## Abstract

**Introduction:**

This study addresses a key gap in climate communication research by examining the differential effects of problem-oriented versus solution-oriented climate narratives on pro-environmental behavioral intention (PEBI) and the underlying mechanisms.

**Methods:**

We conducted two online survey experiments (n1 = 524; n2 = 1,130) to systematically investigate the relationships among narrative strategy, environmental self-efficacy, narrator, and PEBI.

**Results:**

The findings reveal that solution-oriented narratives are significantly more effective than their problem-oriented counterparts in promoting PEBI. Mediation analysis further demonstrates that environmental self-efficacy partially mediates this relationship. In addition, moderated mediation analyses indicate that narrator moderates the effect of narrative strategy on environmental self-efficacy. The positive indirect effect of solution-oriented, relative to problem-oriented, narratives on PEBI through environmental self-efficacy was significant when the central government was the narrator but not when a local government was the narrator.

**Discussion:**

Results advance our understanding of how narratives shape PEBI and provide practical implications for designing more effective climate communication.

## 1 Introduction

Climate change has already emerged as one of the most pressing challenges confronting humanity. A growing body of scholarship suggests that a central strategy for mitigating this challenge lies in the construction and dissemination of effective climate narratives that can mobilize citizens toward greater policy support and more sustainable behaviors (Moezzi et al., 2017; Veland et al., 2018). Appropriately framed, such narratives have the capacity to motivate, empower, explain, coordinate, legitimize, and persuade, thereby enabling the public to recognize their agency in addressing climate change and fostering behavioral change (Bushell et al., 2017).

Media and popular culture frequently frame climate change through “doom and disaster” narratives, portraying it as an existential threat to human society, the natural world, and the planet itself (De Meyer et al., 2021). This approach is conceptualized as a problem-oriented climate narrative, as its defining feature lies in the emphasis on catastrophic losses and profound risks (Said and Wölfl, 2025). Within this narrative frame, climate change is often depicted as an overwhelming challenge and threat.

Research suggests that narratives emphasizing the threat of climate change can make the issue more salient and, under certain conditions, heighten public concern, perceptions of climate risk, and action intentions (Nadeau et al., 2024; O'Neill and Nicholson-Cole, 2009). Nevertheless, such narratives also carry potential drawbacks: they may evoke feelings of helplessness and alienation and, in some cases, induce climate anxiety, ultimately impeding rather than facilitating effective individual or collective action (Feldman and Hart, 2021; Said and Wölfl, 2025).

In recent years, scholars have increasingly advocated a strategic shift toward solution-oriented climate narratives. They argue that governments and communicators should prioritize constructing and disseminating stories that emphasize tangible solutions, rather than focusing on the challenges of climate change (Husain-Naviatti, 2025; Merkel et al., 2020; Troy, 2025). Presenting clear, actionable solutions and linking them to broader, meaningful objectives can significantly influence audiences (Moser, 2010). By doing so, communicators enable recipients to “see the possibility of participation and change,” thereby fundamentally transforming their role from “disengaged bystanders” into “involved participants” (Dales et al., 2024). Such narrative reframing is considered essential for promoting climate-friendly behavior (Mai and von Sikorski, 2025).

Although scholars increasingly emphasize the power of climate narratives, experimental evidence comparing their effects remains limited, and existing findings are mixed. One possible explanation is that previous research has devoted limited attention to how the narrator shapes the effectiveness of different climate narrative strategies. Research has shown that a narrator’s identity and authority, as well as the audience’s trust in the narrator, may influence a narrative’s persuasive and mobilizing effects (Hand et al., 2023; Shanahan et al., 2026). Narrator identity may therefore shape the effectiveness of different climate narrative strategies. However, to date, few studies have examined whether the effects of problem-oriented and solution-oriented climate narratives vary depending on the narrator. This gap limits our understanding of the conditions under which different climate narrative strategies are effective. To address this gap, this study employs an experimental design to systematically examine the effects of problem-oriented and solution-oriented climate narratives on PEBI. Specifically, we compare the effects of the two narrative strategies on PEBI and test both the mediating role of environmental self-efficacy and the moderating role of narrator. This research aims to provide empirical evidence for understanding how climate narratives can effectively motivate public participation, thereby offering a critical contribution to the existing literature.

## 2 Literature review and research hypotheses

### 2.1 Literature review

Narratives and storytelling are vital to how humans live their lives (Bruner, 1987). The ways we perceive and make sense of the world are often shaped by the stories and narratives we tell (Bruner, 1991). Such stories are not merely reflections of the past and the present; they also continuously construct and reshape our understanding of the world (Bruner, 2009; Hardy, 1968; Mertova and Webster, 2019). Narrative communication is particularly important in the context of climate change, which is often perceived as complex, distant, and abstract. By embedding climate facts within a storyline, climate narratives can make the issue more concrete, emotionally meaningful, and relevant to individual behavior (Bevan et al., 2020; Fisher, 1984).

Extant scholarship identifies “problem-oriented” and “solution-oriented” narratives as the predominant strategic paradigms in climate communication (Randall, 2009). Problem-oriented narratives emphasize the losses and risks associated with climate change. A familiar example is the “habitat loss” narrative symbolized by the iconic image of a solitary polar bear, which dramatizes the global climate crisis (Manzo, 2010). By contrast, solution-oriented narratives organize stories around response actions, feasible approaches, implementation pathways, and possible improvements (De Meyer et al., 2021; Thier and Wu, 2025). In China, for example, policy narratives surrounding the goals of carbon peaking and carbon neutrality often emphasize the policies, measures, and actions required to mitigate climate change.

The practical distinction between the two strategies becomes clearer when they are used to narrate the same climate issue. When addressing climate-related drought, for example, a problem-oriented narrative may foreground declining crop yields, water scarcity, and associated economic and livelihood losses. A solution-oriented narrative may instead describe how farmers, communities, and governments adopt water-efficient irrigation practices, drought-resistant crop varieties, and integrated water-management measures to reduce these losses and strengthen climate resilience (Begum et al., 2022). The core difference therefore lies in the dominant focus of the storyline: problem-oriented narratives organize events primarily around the development and consequences of the problem, whereas solution-oriented narratives organize them around actions and pathways for addressing the problem. These distinctions provide the conceptual basis for the present study. Building on this foundation, we systematically examine the differential effects of the two narrative strategies and the potential mechanisms underlying these effects. This approach helps clarify how and under what conditions climate narrative strategies promote PEBI and provides empirical evidence to inform more effective climate communication.

### 2.2 Narrative strategies and pro-environmental behavioral intention

Problem-oriented narratives in climate change communication primarily emphasize loss and risk (Hulme, 2007), including biodiversity loss, reduced crop yields, livelihood insecurity, water scarcity, restrictions on freedom, and large-scale migration (Muluneh, 2021; Porcuna-Ferrer et al., 2024). Extensive research indicates that loss-oriented messages elicit strong emotional responses, such as heightened risk perception and fear, which can act as significant drivers of behavioral change (Heeren et al., 2022; Innocenti et al., 2023; Smith and Leiserowitz, 2014; Van Valkengoed et al., 2024). Other studies suggest that the relationship between hope-related messages and pro-environmental motivations is relatively weak and that their effects are often overshadowed by gloomy messages (Hornsey and Fielding, 2016).

Nevertheless, the effectiveness of problem-oriented narratives has been increasingly questioned. Scholars argue that this narrative may exacerbate feelings of helplessness, anxiety, and fear, thereby undermining both psychological well-being and individual agency (Pihkala, 2020; Reser et al., 2014). Recent scholarship has advocated adopting solution-oriented narratives as an alternative to the long-dominant problem-oriented narratives. Solution-oriented narratives highlight practical strategies and success stories at the individual, organizational, and policy levels, stressing the feasible interventions humans can undertake to address climate change and their positive outcomes. This narrative strategy enhances readers’ sense of agency, thereby counteracting the helplessness often induced by problem-oriented narratives (Said and Wölfl, 2025). Moreover, solution-oriented narratives appear more persuasive among audiences skeptical of governmental climate action, as they emphasize human capacity to confront the crisis (Attari et al., 2010), and have been shown to be more effective in fostering pro-environmental behaviors (Neef et al., 2023).

Based on this review of competing perspectives regarding the two narrative strategies, the present study advances the following competing hypotheses:

*H1*: Climate narratives exert significantly different effects on PEBI.

*H1a*: Compared with solution-oriented narratives, problem-oriented narratives are more effective in promoting PEBI.

*H1b*: Compared with problem-oriented narratives, solution-oriented narratives are more effective in promoting PEBI.

### 2.3 The mediating role of environmental self-efficacy

Self-efficacy is one of the core concepts in social cognitive theory, defined by Albert Bandura as “*the belief in one’s capabilities to organize and execute the courses of action required to produce given attainments*” (Bandura, 1977). It refers to individuals’ belief in their ability to effectively organize and implement behaviors necessary to achieve specific goals. Perceived self-efficacy is generally regarded as a prerequisite for behavior and intention: the higher an individual’s level of self-efficacy, the greater their confidence, competence, and motivation to act when faced with specific tasks (Bandura, 1997). Studies have found that environmental self-efficacy is a key predictor of pro-environmental behavior and intention (Al Mamun et al., 2025; Jugert et al., 2016; Milfont and Sibley, 2012). Individuals with higher levels of environmental self-efficacy are more confident in making sustainable choices (e.g., recycling, green purchasing, or sustainable travel) (Han et al., 2025; Tabernero and Hernández, 2011). Moreover, self-efficacy has been identified as a mediator of the “spillover” from low-cost, easy behaviors to more impactful, demanding pro-environmental actions (Lauren et al., 2016).

According to Bandura (1977), self-efficacy is shaped by four main sources: performance accomplishments, vicarious experiences, verbal persuasion, and emotional arousal. Among these, vicarious experience plays a particularly important role: individuals rely not only on their own successes but also derive efficacy beliefs from observing others successfully accomplishing challenging tasks, which fosters the belief that they too can succeed with sufficient effort (Bandura, 1977). Related research suggests that messages of hope can strengthen self-efficacy by signaling that individual actions can have a meaningful impact, whereas pessimistic messages may overwhelm recipients and diminish efficacy beliefs (Huang and Guo, 2024). Similarly, studies in the field of communication have shown that exposure to constructive or solution-oriented news can enhance perceived self-efficacy by providing vicarious information and modeling effects, thereby fostering individuals’ capacity for action (Overgaard, 2023).

In summary, these findings suggest that compared to problem-oriented climate narratives, solution-oriented narratives are more likely to enhance individuals’ environmental self-efficacy and, in turn, their willingness to engage in pro-environmental behaviors. Accordingly, we propose the following hypothesis:

*H2*: Environmental self-efficacy mediates the relationship between climate narratives and PEBI. Compared with problem-oriented narratives, solution-oriented narratives are more effective in enhancing individuals’ environmental self-efficacy, which in turn promotes their PEBI.

### 2.4 The moderating role of narrators

The NPF posits that a story’s success is not just about its content; it’s about who tells it (Jones and McBeth, 2010). An influential narrative must be credible, come from a trustworthy source, and resonate with the audience’s existing identity (Hajer, 1995; László, 2008). This is particularly crucial for climate narratives, as it helps us understand why different messengers telling the same story can have vastly different outcomes. As trusted information sources and shapers of social norms, governments play a central role in climate communication (Ferranti et al., 2021). By articulating the causes, impacts, future trajectories, and response strategies of climate change, governments help shape the perceived legitimacy of climate policies and foster broader societal engagement (Zhang and Orbie, 2021).

Bandura (1977) identifies “persuasion” as a key dimension shaping individuals’ beliefs in their own capabilities, noting that the credibility, reputation, and trustworthiness of the persuader can substantially influence the audience’s sense of self-efficacy. When the source of information is perceived as trustworthy, individuals are more inclined to believe that they can take effective action. Empirical evidence lends support to this proposition. Kim and Doh (2022) demonstrate that higher levels of public trust in government are positively associated with citizens’ willingness to accept government-provided disaster preparedness knowledge and training, thereby strengthening their confidence in crisis response. Similarly, Hong et al. (2025) find that when individuals perceive the government as capable of effectively addressing threats such as drought or climate change, their self-efficacy increases significantly, which in turn promotes proactive adaptive behaviors.

In China, the stratified structure of government trust may further condition the relationship between trust and self-efficacy. Research indicates a significant difference in public trust across government levels, with the central government consistently viewed as having higher authority and credibility than local governments, creating a pattern of “hierarchical governmental trust” (*chaxuxinren*) (Li, 2016; Zhan et al., 2025; Zhou, 2007). This trust asymmetry carries important implications for climate communication. Individuals who perceive the central government as more credible are, by extension, more likely to internalize climate narratives originating from central authorities, thereby experiencing a stronger enhancement of self-efficacy and, consequently, greater PEBI. By contrast, the comparatively lower trust accorded to local governments may attenuate the persuasive effect of their climate narratives on public self-efficacy. Based on these insights, we propose the following hypotheses:

*H3a*: The narrator significantly moderates the relationship between the climate narrative strategy and environmental self-efficacy.

*H3b*: The indirect effect of climate narrative strategy on PEBI through environmental self-efficacy is stronger when the central government serves as the narrator than when the local government serves as the narrator.

## 3 Experiment 1

### 3.1 Experimental design

This study employed a two-phase experimental approach to test the aforementioned hypotheses. The first experiment aimed to achieve two primary objectives: (1) to compare the impact of problem-oriented versus solution-oriented climate narratives on PEBI, and (2) to examine the mediating role of self-efficacy in the relationship between narrative strategy and PEBI. To this end, we conducted an online, between-subjects experimental survey. The intervention involved exposing participants to one of two distinct climate narratives.

### 3.2 Participants

The target population for this study consists of participants from China. Utilizing a Chinese sample is of significant academic value for enhancing both the external validity and the contextual richness of climate communication research. On the one hand, as a major developing economy confronting severe climate risks—such as persistent smog and extreme weather events—China provides a unique perspective for examining the effects of climate narratives on public environmental intentions and behaviors within a Global South governance framework. On the other hand, the inclusion of Chinese participants helps address the current under-representation of non-Western contexts in climate narrative scholarship and provides a critical cross-cultural validation for narrative persuasion theories.

The experiment was conducted via Credamo^1^, a professional data collection platform widely utilized in Chinese social science research. Participants were recruited from the platform’s respondent pool and received compensation of approximately RMB 3 upon successful completion of the survey. Participants were randomly assigned to experimental conditions using the platform’s randomization function. An *a priori* power analysis was conducted with G*Power to determine the required sample size for a one-way ANOVA with two groups. Parameters were set to a medium effect size (*f* = 0.25), a statistical power of 0.95, and a significance level of 0.05, based on Tai et al. (2024). The analysis indicated that at least 210 participants were necessary.

Experiment 1 included 524 participants, with 278 assigned to the problem-oriented narrative group and 246 to the solution-oriented narrative group. Descriptive statistics are presented in Table 1. Both groups had a similar mean age of approximately 31 years. In the problem-oriented group, 28.8% of participants were male and 71.2% were female, while the solution-oriented group comprised 35.0% male and 65.0% female participants. The majority of participants in both groups held a bachelor’s degree (67.3 and 73.2%, respectively) and resided in urban areas (91.0 and 90.2%, respectively). In both groups, most participants in both groups reported one to five instances of prior environmental behavior.

**Table 1:** Descriptive statistics of survey participants in experiment 1.

Variable	Treatment group		*χ* ^2^	*p*
	Problem-oriented	Solution-oriented		
*N*	278	246		
Gender [%]: Male	28.8%	35.0%	2.305	0.129
Female	71.2%	65.0%		
Age	30.65	31.48	2.425	0.658
Education [%]: ≤Junior High	1.4%	1.2%	2.197	0.699
High School	3.6%	2.8%		
Associate	11.5%	9.8%		
Bachelor’s	67.3%	73.2%		
≥Master’s	16.2%	13.0%		
Residence [%]: Urban	91.0%	90.2%	0.022	0.881
Rural	9.0%	9.8%		
Environmental behavior experience: Never	2.5%	3.1%	7.178	0.127
1 ~ 2 times	45.3%	36.2%		
3 ~ 5 times	34.6%	48.5%		
≥6 times	17.6%	12.3%		

Age was categorized into five groups for the chi-square tests: ≤24, 25–34, 35–44, 45–54, and ≥55 years. Mean age is reported in the table for descriptive purposes.

To confirm the effectiveness of the random assignment, chi-square tests were conducted across all demographic variables. As shown in the final two columns of Table 1, none of the *p*-values were statistically significant. This indicates that the two experimental groups were well-balanced and comparable across key demographic characteristics, including gender (*χ*^2^ = 2.305, *p* = 0.129), age (*χ*^2^ = 2.425, *p* = 0.658), education (*χ*^2^ = 2.197, *p* = 0.699), residence (*χ*^2^ = 0.022, *p* = 0.881), and prior environmental behavior experience (*χ*^2^ = 7.178, *p* = 0.127).

### 3.3 Stimulus materials

The experimental stimuli consisted of two written narratives, both centered on the theme of climate change. Following the view by Shanahan et al. (2011) that *“policy narratives are pervasive in the policy arena, including interest group letters, speeches, and media reports*,” the stimulus materials were adapted from an official climate report published by *People’s Daily*^2^.

In terms of structural design, both narrative conditions followed a three-part format: background, core manipulation, and reinforcement (Said and Wölfl, 2025). The first paragraph provided all participants with identical introductory information outlining the causal link between human activities and climate change, establishing a uniform cognitive foundation prior to the narrative intervention. The second paragraph constituted the core experimental manipulation. In the problem-oriented condition, the narrative emphasized the adverse consequences and potential risks associated with climate change; in the solution-oriented condition, it highlighted positive policy outcomes and actionable mitigation measures. The third paragraph served as the concluding section, reinforcing the respective narrative orientation established in the preceding paragraph. Across conditions, the total length of each narrative was controlled at approximately 465 Chinese characters. The full texts of all stimulus materials are provided in Appendix.

### 3.4 Measures

The PEBI was assessed using three items adapted from Stern (2000). The items were contextualized to measure participants’ behavioral intentions following exposure to the experimental materials: (1) “I am willing to actively conserve water and electricity in my daily life”; (2) “I am willing to choose products with minimal or recyclable packaging when shopping, even if it costs more”; and (3) “I am willing to invest more time and effort in sorting and recycling waste in my daily life.” These items were designed to capture individuals’ willingness to engage in pro-environmental behaviors in their daily routines and consumption choices. Responses were recorded on a 7-point Likert scale (1 = strongly disagree, 7 = strongly agree). The scale demonstrated good internal consistency (Cronbach’s *α* = 0.82).

Environmental self-efficacy was measured using three items adapted from the Self-Efficacy Scale developed by Yoong et al. (2018). The specific items included: (1) “I can practice pro-environmental activities very easily”; (2) “I think that my ability to adopt a pro-environmental lifestyle is greater than others”; and (3) “I am able to understand the ways of sustaining the environment and apply them effectively.” The scale demonstrated good internal consistency (Cronbach’s *α* = 0.79).

### 3.5 Procedure

The study was pre-registered on the Open Science Framework (registration doi: 10.17605/OSF.IO/32PZV) prior to data collection and was approved by the Ethics Committee of Northeastern University. Furthermore, all participants provided voluntary consent through an informed consent form that clearly stated their right to withdraw from the study at any time and the measures taken to protect their privacy.

Following consent, participants were presented with the experimental stimulus materials. Those assigned to the two treatment groups read climate narratives, either problem-oriented or solution-oriented. The narratives were presented as written texts. To encourage attentive reading, a countdown timer was included on the experiment page, requiring participants to remain on the page for a minimum duration before proceeding.

To verify the effectiveness of the manipulation without disrupting the flow of the experiment, participants completed a brief manipulation check immediately after reading the materials. The check consisted of two items: “I think this material primarily emphasizes climate change problems” and “I think this material primarily emphasizes climate response solutions.” Both items were rated on a 7-point Likert scale (1 = strongly disagree, 7 = strongly agree). In addition, three attention check items were embedded at various points throughout the experiment to ensure data quality.

### 3.6 Results

#### 3.6.1 Manipulation check

To confirm the effectiveness of the narrative manipulation, we conducted independent-samples *t*-tests on participants’ perceptions of the problem-oriented and solution-oriented features of the materials. On the problem-oriented perception item, participants in the problem-oriented condition reported significantly higher scores (*M* = 4.50, SD = 2.41) than those in the solution-oriented condition (*M* = 1.43, SD = 0.71), *t*(522) = 19.25, *p* < 0.001, mean difference = 3.07, 95% CI[2.76, 3.38]. Conversely, on the solution-oriented perception item, participants in the solution-oriented condition reported significantly higher scores (*M* = 6.61, SD = 0.68) than those in the problem-oriented condition (*M* = 3.85, SD = 2.55), *t*(522) = 16.46, *p* < 0.001, mean difference = 2.76, 95% CI[2.43, 3.09]. These findings confirm that the manipulation of narrative strategy was successful in Experiment 1.

#### 3.6.2 Testing for hypothesis 1

To test our primary hypothesis, we first conducted a Levene’s test for equality of variances on the PEBI scores of the two groups. The results in Table 2 indicated a significant difference in variance between the problem-oriented and solution-oriented narrative groups (*F* = 3.998, *p* = 0.046). Accordingly, a two-tailed independent samples t-test assuming unequal variances was used for hypothesis testing. The *t*-test results revealed a significant difference in PEBI scores between the two groups, *t*(521.44) = −4.545, *p* < 0.001. Specifically, the solution-oriented narrative group showed significantly higher PEBI (*M* = 6.145, SD = 0.719) compared to the problem-oriented narrative group (*M* = 5.835, SD = 0.840). This finding provides strong evidence supporting our core hypothesis that solution-oriented narratives are more effective than problem-oriented narratives in promoting PEBI. Consequently, H1 and H1b were supported, while H1a was rejected.

**Table 2:** Main effect analysis results.

Group	*N*	Mean	SD	Levene *F*	Levene *p*	*t*	*p*
Problem-oriented	278	5.835	0.840	3.998	0.046	−4.545	<0.001***
Solution-oriented	246	6.145	0.719				

***p < 0.001.

#### 3.6.3 Testing for hypothesis 2

To test the mediating role of environmental self-efficacy between narrative strategy and PEBI, we used Model 4 of the PROCESS macro. The analysis was conducted using version 4.2 of the PROCESS macro in SPSS 26.0. Age, gender, education, residence, and environmental behaviors experience were included as covariates. The indirect effect was estimated using 5,000 bootstrap samples with a 95% confidence level.

**Figure 1:**
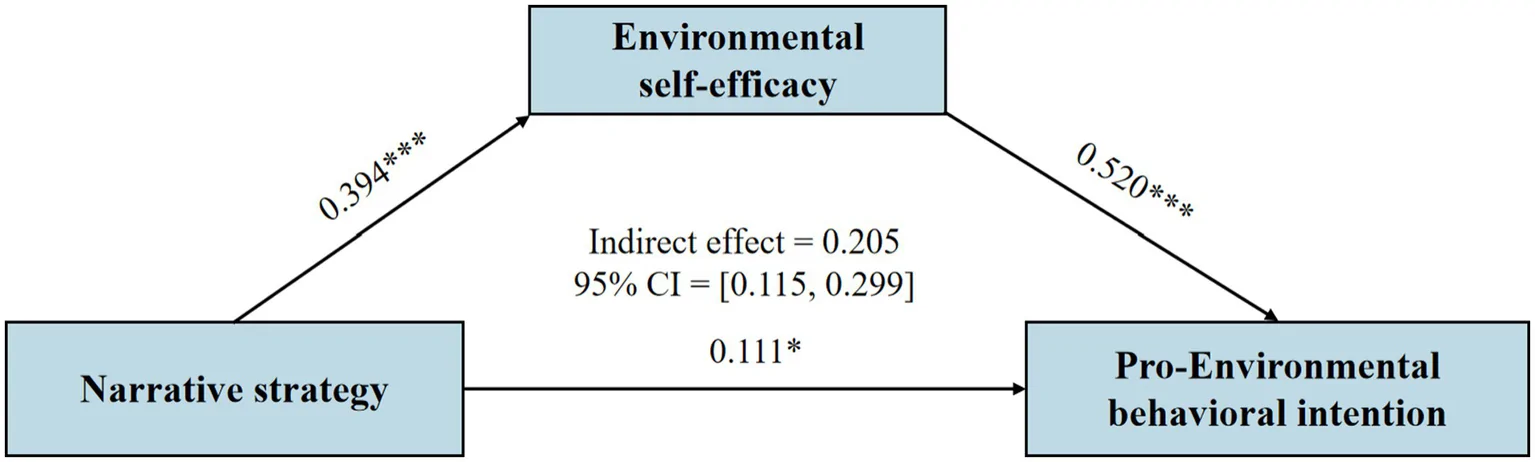
Mediation model of the effect of narrative strategy on PEBI through environmental self-efficacy. *p < 0.05, ***p < 0.001.

As presented in Table 3 and Figure 1, the mediation analysis revealed that narrative strategy significantly predicted environmental self-efficacy (*b* = 0.394, SE = 0.085, *p* < 0.001), with the solution-oriented condition associated with higher self-efficacy than the problem-oriented condition. Environmental self-efficacy, in turn, positively predicted PEBI (*b* = 0.520, SE = 0.027, *p* < 0.001). The indirect effect through environmental self-efficacy was significant (indirect effect = 0.205, BootSE = 0.047, 95% bootstrap CI [0.115, 0.299]), providing evidence for the mediating role of environmental self-efficacy. The mediation model and corresponding path coefficients are illustrated in Figure 1. The direct effect of narrative strategy on PEBI remained significant but was reduced in the model (*b* = 0.111, SE = 0.054, *p* = 0.040), indicating partial mediation. These findings suggest that, compared with problem-oriented narratives, solution-oriented narratives enhance environmental self-efficacy, which, in turn, is associated with stronger PEBI. Thus, H2 was supported.

**Table 3:** Results of mediation analysis.

Path	Effect	SE/Boot SE	*p*	LL CI	UL CI
Narrative strategy → Environmental self-efficacy	0.3941	0.0851	<0.001***	0.2269	0.5612
Environmental self-efficacy → PEBI	0.5202	0.0272	<0.001***	0.4667	0.5736
Narrative strategy → PEBI (Total effect)	0.3156	0.0687	<0.001***	0.1807	0.4506
Narrative strategy → PEBI(Direct effect)	0.1107	0.0537	0.040*	0.0052	0.2161
Narrative strategy → Environmental self-efficacy → PEBI (Indirect effect)	0.2050	0.0465		0.1152	0.2991

BootSE is bootstrap standard error based on 5,000 samples. Indirect effect significance was determined using 95% bootstrap confidence intervals. *p < 0.05, **p < 0.01, ***p < 0.001.

## 4 Experiment 2

### 4.1 Experiment design

Experiment 2 was designed to further explore the mechanisms of climate narratives, specifically investigating the moderating role of the narrator. The primary objective was to determine whether the effectiveness of two narrative strategies differs depending on the government level acting as the source. To achieve this, we employed a 2 (Narrator: central vs. local Government) × 2 (Narrative Strategy: problem-oriented vs. solution-oriented) between-subjects experimental design, which resulted in four distinct experimental conditions. For consistency, the measurement tools for PEBI and environmental self-efficacy were identical to those used in Experiment 1.

### 4.2 Participants

Experiment 2 also recruited participants via the Credamo online survey platform. All participants were randomly assigned to one of the four experimental conditions using the platform’s randomization function. A total of 1,130 individuals were recruited for this experiment. Table 4 provides the descriptive statistics for all experimental groups in Experiment 2.

**Table 4:** Descriptive statistics of survey participants in Experiment 2.

Variable	Treatment group				*χ* ^2^	*p*
	Problem-oriented		Solution-oriented			
	Central	Local	Central	Local		
*N*	244	295	250	341		
Gender [%]: Male	24.6%	25.8%	28.0%	27.3%	0.933	0.818
Female	75.4%	74.2%	72.0%	72.7%		
Age	31.27	30.67	32.02	30.75	12.833	0.381
Education [%]: ≤Junior High	2.5%	0.7%	0.8%	0.9%	9.431	0.666
High School	2.0%	2.7%	2.8%	2.1%		
Associate	11.1%	10.8%	12.0%	8.5%		
Bachelor’s	69.3%	72.2%	70.4%	71.0%		
≥Master’s	15.2%	13.6%	14.0%	17.6%		
Residence [%]: Urban	93.0%	91.5%	91.6%	91.8%	0.511	0.916
Rural	7.0%	8.5%	8.4%	8.2%		
Environmental behavior experience [%]: Never	10.2%	11.9%	10.0%	10.6%	10.918	0.281
1 ~ 2 times	66.8%	70.2%	64.8%	67.2%		
3 ~ 5 times	14.8%	7.1%	14.0%	12.6%		
≥6 times	8.2%	10.8%	11.2%	9.7%		

Age was categorized into five groups for the chi-square tests: ≤24, 25–34, 35–44, 45–54, and ≥55 years. Mean age is reported in the table for descriptive purposes.

To ensure the effectiveness of the randomization process, chi-square tests were conducted on all demographic variables across the four experimental groups. The results showed no statistically significant differences in any of the variables (all *p* > 0.05). Specifically, there were no significant differences between the groups in terms of gender (*χ*^2^ = 0.933, *p* = 0.818), age (*χ*^2^ = 12.833, *p* = 0.381), education (*χ*^2^ = 9.431, *p* = 0.666), residence (*χ*^2^ = 0.511, *p* = 0.916), and environmental behavior experience (*χ*^2^ = 10.918, *p* = 0.281). This confirms that our random assignment was successful in creating a well-balanced sample across key demographic characteristics.

### 4.3 Stimulus materials

Experiment 2 aimed to examine the moderating role of narrator identity. Building on the narratives used in experiment 1, we introduced an additional manipulation of the source. To ensure that the institutional origin of the narrative was salient before participants engaged with the content, each text was prefaced by a brief, bolded source statement rather than a closing byline. In the central government condition, the narrative was introduced with the statement, “The following is a climate response report released by a central government department”; in the local government condition, it was introduced with, “The following is a climate response report released by a provincial people’s government.” The four versions of the stimulus materials were kept consistent in structure, length, vocabulary, and writing style, differing only in narrative orientation and source attribution.

### 4.4 Results

#### 4.4.1 Manipulation check

To ensure the effectiveness of our experimental manipulation, we conducted a series of checks on narrative strategy (problem-oriented vs. solution-oriented) and narrator (central vs. local government). We also assessed the perceived accuracy of the materials across all experimental conditions.

Independent-samples t-tests revealed that participants in the problem-oriented condition rated the materials as significantly more problem-oriented than did those in the solution-oriented condition (*M* = 6.52, SD = 0.72 vs. *M* = 1.36, SD = 0.73; *t*(1128) = 119.39, *p* < 0.001). Participants in the solution-oriented condition rated the materials as significantly more solution-oriented than did those in the problem-oriented condition (*M* = 6.48, SD = 0.73 vs. *M* = 1.52, SD = 0.86; *t*(1062.85) = −104.13, *p* < 0.001). These results confirm that the manipulation of narrative strategy was effective.

Similarly, participants in the central government condition perceived the narrator as significantly more representative of central government than did those in the local government condition (*M* = 6.52, SD = 0.67 vs. *M* = 1.31, SD = 0.64; *t*(1128) = 134.22, *p* < 0.001). The reverse pattern was observed for the local government perception dimension (*M* = 6.53, SD = 0.73 vs. *M* = 1.42, SD = 0.70; *t*(1128) = −118.70, *p* < 0.001). These findings confirm that the narrator manipulation was also effective.

To verify that the two manipulated factors operated independently, we examined whether narrator identity affected perceptions of narrative strategy. Within the problem-oriented condition, the central and local government groups did not differ significantly on either the problem-oriented perception dimension [*t*(537) = −1.04, *p* = 0.298] or the solution-oriented perception [*t*(537) = −1.13, *p* = 0.258]. Within the solution-oriented condition, a similar pattern was observed: the groups did not differ on problem-oriented perception [*t*(589) = −0.21, *p* = 0.835] or solution-oriented perception [*t*(589) = 1.45, *p* = 0.148].

An ANOVA on perceived accuracy revealed no significant main effects or interaction (all *F*s < 1, *p*s > 0.05). Across all four conditions, participants rated the materials as highly accurate (*M* = 6.09, SD = 1.16), indicating that the stimulus materials were equivalent in perceived informational quality regardless of experimental condition.

#### 4.4.2 Testing for hypothesis 3

To test hypotheses H3a and H3b, we employed PROCESS Model 7 and estimated the conditional indirect effects using 5,000 bootstrap samples. As shown in Table 5, the interaction between narrative strategy and narrator was statistically significant (*b* = −0.260, SE = 0.077, *t* = −3.366, *p* < 0.001, 95% CI[−0.411, −0.108]), indicating that narrator identity significantly moderated the effect of narrative strategy on environmental self-efficacy.

**Table 5:** Results of moderated mediation analysis.

Effect	*b*	SE	*t*	*p*	LLCI	ULCI
Outcome: Environmental self-efficacy						
Narrative strategy	0.270	0.058	4.667	<0.001	0.157	0.384
Narrator (0 = Central, 1 = Local)	−0.019	0.056	−0.337	0.736	−0.128	0.090
Narrative strategy × Narrator	−0.260	0.077	−3.366	<0.001	−0.411	−0.108
Conditional Effects						
Central gov (0)	0.270	0.058	4.667	<0.001	0.157	0.384
Local gov (1)	0.010	0.051	0.203	0.839	−0.090	0.111
Outcome: PEBI						
Environmental self-efficacy	0.618	0.027	22.721	<0.001	0.564	0.671
Narrative strategy (direct effect)	−0.110	0.035	−3.091	0.002	−0.179	−0.040

The model controls for age, gender, education, residence, and environmental behavior experience.

**Figure 2:**
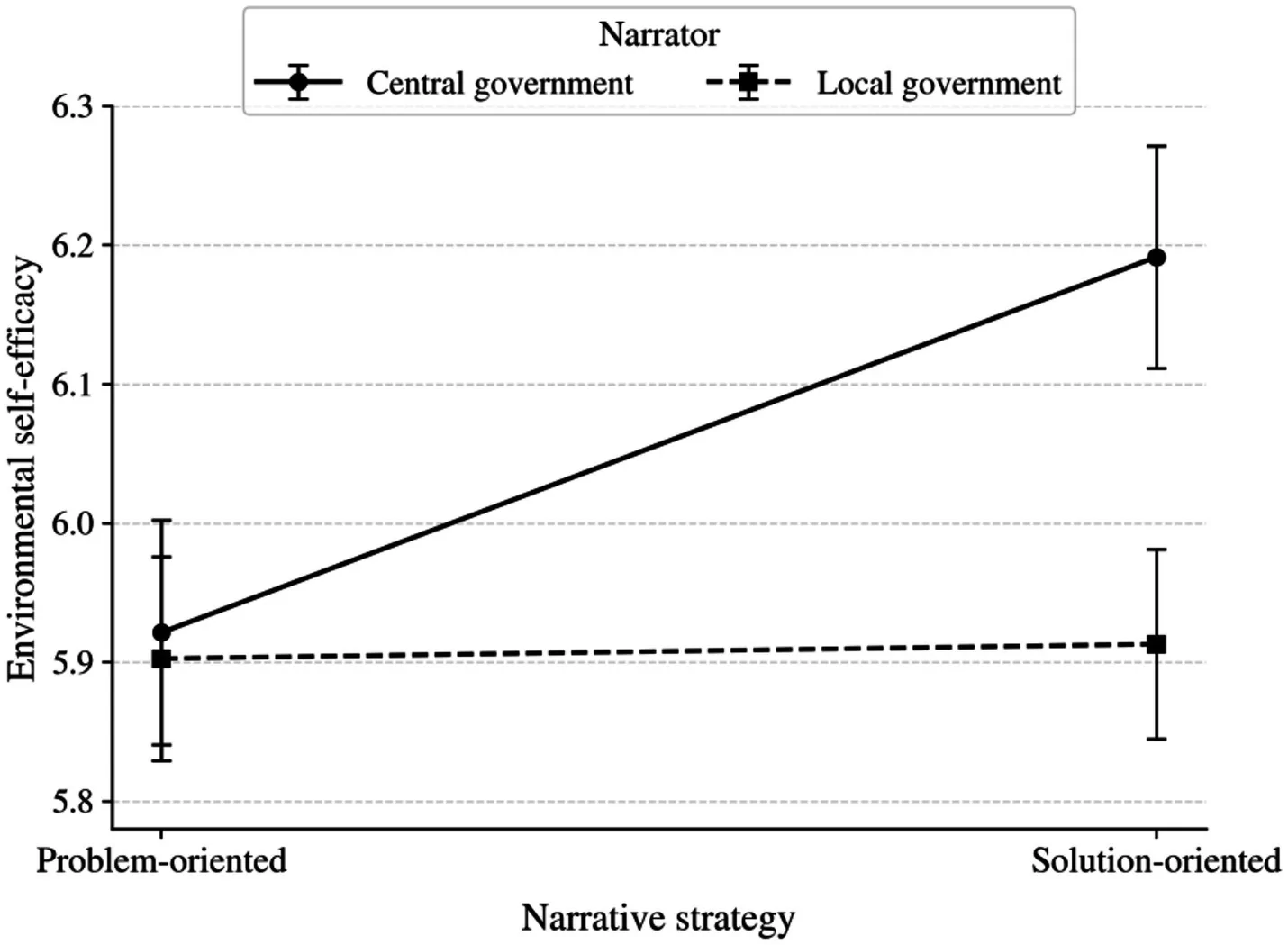
Interaction between narrative strategy and narrator on environmental self-efficacy.

Specifically, when the narrator was the central government, the effect of narrative strategy on environmental self-efficacy was significant (*b* = 0.270, SE = 0.058, *p* < 0.001, 95% CI[0.157, 0.384]). By contrast, when the narrator was the local government, the effect was not significant (*b* = 0.010, SE = 0.051, *p* = 0.839, 95% CI[−0.090, 0.111]). As illustrated in Figure 2, solution-oriented narratives increased environmental self-efficacy when the narrator was the central government, whereas this effect was not observed when the narrator was the local government. Thus, H3a was supported.

**Figure 3:**
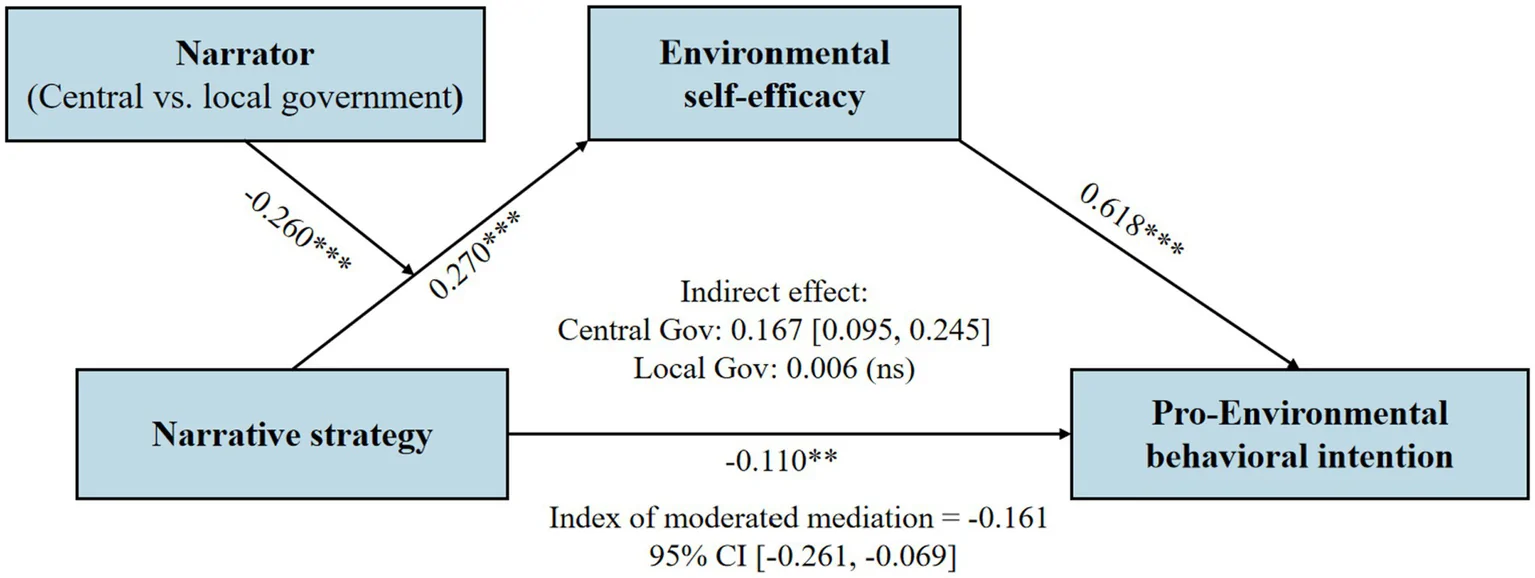
Moderated mediation effect of narrator on the relationship between narrative strategy and PEBI. **p < 0.01, ***p < 0.001.

We further examined whether narrator moderated the indirect effect of narrative strategy on PEBI through environmental self-efficacy. As shown in Table 6, the index of moderated mediation was statistically significant (index = −0.161, BootSE = 0.049, 95% bootstrap CI[−0.261,−0.069]), as the confidence interval did not include zero. The moderated mediation model and the corresponding conditional indirect effects are illustrated in Figure 3. This finding indicates that the mediating role of environmental self-efficacy is indeed significantly moderated by the narrator. Specifically, when the central government was the narrator, the indirect effect of the solution-oriented condition, compared with the problem-oriented condition, on PEBI through environmental self-efficacy was significant (indirect effect = 0.167, BootSE = 0.038, 95% bootstrap CI [0.095, 0.245]). This indirect effect was not significant when the local government was the narrator (indirect effect = 0.006, BootSE = 0.031, 95% bootstrap CI [−0.054, 0.067]). Thus, H3b was supported.

**Table 6:** Conditional indirect effects of narrative strategy on PEBI through self-efficacy at different levels of narrator.

Narrator	Indirect effect	BootSE	95% CI LL	95% CI UL
Central Government (narr = 0)	0.167	0.038	0.095	0.245
Local Government (narr = 1)	0.006	0.031	−0.054	0.067
Index of Moderated Mediation	−0.161	0.049	−0.261	−0.069

## 5 General discussion

How we tell the story of climate change fundamentally shapes our collective capacity to understand and adapt to it. For decades, risk discourse has dominated climate communication. In recent years, a growing body of research has emphasized the importance of climate narratives that highlight concrete actions and solutions. As Betz (2015) argues, an effective strategic narrative should clearly articulate “what is being done and why.” Despite this growing emphasis, empirical evidence remains limited regarding whether solution-oriented narratives are more effective than problem-oriented narratives in promoting PEBI. Therefore, our study addresses a gap in the literature by systematically comparing the effects of problem-oriented versus solution-oriented climate narratives on PEBI and by examining the mechanisms through which these effects occur.

First, we extend the focus of climate narrative research from the effects of narrative elements to comparisons between narrative strategies. Previous studies have primarily examined how specific elements, such as narrative characters and features, shape audiences’ understanding of and responses to climate change (Fonseca and Castro, 2026; Yan et al., 2026). The present study examines two narrative strategies often used in climate communication and systematically compares the differential effects of problem-oriented and solution-oriented narratives on PEBI. The results show that solution-oriented narratives are more effective than problem-oriented narratives in promoting PEBI. The potential value of solution-oriented narratives lies in their capacity to help audiences perceive climate change as a solvable problem while strengthening their confidence and motivation to take pro-environmental action. This finding responds to scholarly calls for climate communication to move beyond problem identification toward constructive responses (Chabay et al., 2019; Merkel et al., 2020) and provides new experimental evidence regarding which narrative strategies are more effective in motivating PEBI.

Second, this study identifies environmental self-efficacy as a key psychological mechanism through which climate narrative strategies influence PEBI. Previous research has established environmental self-efficacy as an important predictor of pro-environmental intentions and behaviors (Lauren et al., 2016). However, less attention has been paid to how different climate messages cultivate such efficacy beliefs. Our results show that, compared with problem-oriented narratives, solution-oriented narratives significantly enhance environmental self-efficacy, which in turn increases PEBI, indicating a partial mediating effect. This finding not only reinforces previous research linking efficacy beliefs to climate engagement (Feldman and Hart, 2021; Thier and Lin, 2022), but also demonstrates that environmental self-efficacy constitutes a specific psychological pathway through which climate narratives translate into PEBI. Environmental self-efficacy should therefore be understood not merely as a pre-existing antecedent of PEBI, but also as a narrative-responsive appraisal that can be strengthened by the actionable solutions and pathways conveyed in climate narratives. By identifying this mediating process, the study explains why solution-oriented narratives are more effective in promoting PEBI and extends the theoretical role of environmental self-efficacy in climate narrative persuasion.

Finally, this study identifies narrator as a boundary condition of climate narrative effectiveness. Although the NPF suggests that narrative persuasion depends on both narrative content and narrator identity, the interaction between narrator identity and narrative strategy remains underexplored in existing research. In climate communication, governments are among the most authoritative and central producers of climate narratives. Comparing central and provincial governments as narrators enabled us to examine whether the same narrative strategy may produce different effects when communicated by different institutional actors. The results show that solution-oriented narratives significantly increased environmental self-efficacy relative to problem-oriented narratives when the central government was the narrator, whereas this difference was not significant when a local government was the narrator. These findings indicate that governmental narrator identity does not exert a uniform influence across climate narratives; rather, its effect varies with narrative strategy. One possible explanation is that solution-oriented narratives are particularly sensitive to the perceived authority and credibility of the narrator. Previous research suggests that governmental trust in China is hierarchically differentiated, with the central government generally accorded greater credibility than subnational governments. Solutions and action pathways communicated by the central government may therefore be perceived as more feasible, thereby generating stronger environmental self-efficacy. Problem-oriented narratives, by contrast, require audiences to accept an assessment of risk. While climate risks may include uncertain threats, this type of judgment can be corroborated through multiple channels, including scientific consensus, media coverage, and personal experience of extreme weather events. The availability of these alternative validation pathways reduces the audience’s reliance on any single narrator (Metzger and Flanagin, 2013).

Because this study did not directly measure differences in trust across levels of government, this explanation should be regarded as theoretically informed rather than empirically tested. Nevertheless, the findings support the NPF’s general proposition regarding the importance of narrators and further refine it by demonstrating that narrator effects vary across narrative strategies. In institutional contexts where local governments enjoy higher levels of public trust, the observed moderating pattern may be attenuated or even reversed (Fitzgerald and Wolak, 2016). Overall, the findings suggest that analyses of narrative persuasion should consider not only narrative strategy but also narrator identity and the interaction between them.

## 6 Practical implications

Our findings carry practical implications for governments and policymakers seeking to improve the effectiveness of climate communication. First, climate communicators should recalibrate the relative priority given to different climate narrative strategies. Climate communication has long been dominated by messaging that emphasizes risks, threats, and the severity of the crisis (Lohkamp et al., 2026; Stecula and Merkley, 2019). Our findings show that solution-oriented narratives are more effective in promoting PEBI. Climate communicators should therefore shift greater emphasis from repeatedly highlighting the problem toward presenting concrete actions, implementation pathways, and potential outcomes. This can help audiences perceive climate change as a solvable problem and understand how they can participate in addressing it. This does not mean that problem-oriented narratives should be completely abandoned. An exclusive emphasis on solution-oriented narratives may weaken public perceptions of problem severity and the urgency of action. A more balanced strategy should therefore give greater prominence to solution-oriented narratives while retaining an appropriate level of problem-oriented content. This combination can sustain public attention to climate risks while strengthening confidence and motivation to take action.

Second, policymakers and climate communicators should make strengthening environmental self-efficacy among the public a central objective of solution-oriented narratives. Our findings show that solution-oriented narratives promote PEBI partly by strengthening individuals’ beliefs in their ability to act and in the value of their contributions. Policymakers and climate communicators should translate broad climate goals into concrete and feasible pathways for public participation, clearly demonstrating how individual actions contribute to broader climate outcomes. Practical guidance, relatable examples of citizen participation, and verifiable evidence of behavioral impact can further strengthen this connection (Metag et al., 2016). Moreover, the public should not be portrayed merely as victims of climate change or passive beneficiaries of climate policy, but as active participants whose contributions can make a meaningful difference. Helping individuals recognize both their capacity to participate and the potential impact of their actions can promote environmental self-efficacy and, in turn, PEBI.

Third, climate communication should consider not only what is communicated but also who communicates it, ensuring alignment between narrative strategy and narrator identity. The influence of narrator identity is not uniform across narrative strategies; solution-oriented narratives may be particularly sensitive to the narrator’s authority, perceived credibility, and implementation capacity. Therefore, narratives presenting action pathways, policy commitments, and expected outcomes should be communicated or endorsed by actors with the authority, credibility, and capacity required to implement the proposed actions. Such alignment may strengthen public confidence in the feasibility of proposed solutions and increase motivation to engage in climate action. Different climate narrators should also coordinate their communicative roles according to their respective institutional mandates and capabilities, rather than disseminating repetitive or homogeneous narratives.

## 7 Limitations and future directions

This study has several limitations, which in turn suggest avenues for future research. First, we did not directly measure participants’ relative trust in central versus local government, instead relying on the well-documented pattern of hierarchical trust in China to interpret our findings. Future research incorporating direct measures of multilevel governmental trust, ideally as a moderator within the same experimental framework, would substantially strengthen causal inference about the underlying process.

Second, urban and highly educated respondents were overrepresented in our sample, which may limit the generalizability of the findings. Although education, residence, and other demographic characteristics were included as covariates to improve the robustness of the analyses, statistical adjustment cannot fully compensate for the limited representation of rural and less-educated populations. Our findings therefore provide relatively reliable evidence on how populations with relatively high levels of education respond to different climate narratives, while their applicability to other groups requires further testing using representative samples and cross-group replications.

Third, owing to resource and practical constraints, this study relied primarily on online survey experiments to examine the effects of different climate narrative strategies on PEBI and the underlying psychological mechanisms. Behavioral intentions, however, should not be equated with actual behavior. Nevertheless, PEBI are an important antecedent of actual pro-environmental behavior. By identifying the causal effects and psychological mechanisms through which climate narratives shape such intentions, this study provides a basis for further examining how climate narratives may translate into actual behavior. Given the importance of clarifying the gap between pro-environmental intentions and behavior, future research should combine field experiments, longitudinal designs, and consequential behavioral measures to examine whether narrative effects persist and translate into actual behavior.

## Data Availability

The raw data supporting the conclusions of this article will be made available by the authors, without undue reservation.

## Footnotes

1
www.credamo.com

2 People’s Daily is the official newspaper of the Central Committee of the Communist Party of China, directly overseen by the central government. It is widely regarded as the most authoritative media outlet in China and serves as a primary source of policy information for the public. Its unique combination of political authority and narrative elements makes it a particularly suitable data source for research on climate narratives.

## References

[ref1] Al Mamun A. Yang M. Hayat N. Gao J. Yang Q. (2025). The nexus of environmental values, beliefs, norms and green consumption intention. Hum. Soc. Sci. Commun. 12:634. doi: 10.1057/s41599-025-04979-6

[ref2] Attari S. Z. DeKay M. L. Davidson C. I. de Bruin W. B. (2010). Public perceptions of energy consumption and savings. Proc. Natl. Acad. Sci. USA 107, 16054–16059. doi: 10.1073/pnas.1001509107, 20713724 PMC2941272

[ref3] Bandura A. (1977). Self-efficacy: toward a unifying theory of behavioral change. Psychol. Rev. 84, 191–215. doi: 10.1037/0033-295X.84.2.191, 847061

[ref4] Bandura A. (1997). Self-efficacy: The exercise of control. New York, NY: W. H. Freeman and Company.

[ref5] Begum R. A. Lempert R. Ali E. Benjaminsen T. A. Bernauer T. Cramer W. . (2022). “Point of departure and key concepts,” in Climate Change 2022: Impacts, Adaptation and Vulnerability: Working Group II Contribution to the Sixth Assessment Report of the Intergovernmental Panel on Climate Change, eds. Pörtner H.-O. Roberts D. C. Tignor M. Poloczanska E. S. Mintenbeck K. Alegría A. . (Cambridge, UK and New York, NY, USA: Cambridge University Press), 121–196. doi: 10.1017/9781009325844.003

[ref6] Betz D. (2015). Carnage and Connectivity: Landmarks in the Decline of Conventional Military Power. New York, NY: Oxford University Press.

[ref7] Bevan L. D. Colley T. Workman M. (2020). Climate change strategic narratives in the United Kingdom: emergency, extinction, effectiveness. Energy Res. Soc. Sci. 69:101580. doi: 10.1016/j.erss.2020.101580

[ref8] Bruner J. (1987). Life as narrative. Soc. Res. 54, 11–32.

[ref9] Bruner J. (1991). The narrative construction of reality. Crit. Inq. 18, 1–21. doi: 10.1086/448619

[ref10] Bruner J. S. (2009). Actual Minds, Possible Worlds. Cambridge, MA: Harvard University Press.

[ref11] Bushell S. Buisson G. S. Workman M. Colley T. (2017). Strategic narratives in climate change: towards a unifying narrative to address the action gap on climate change. Energy Res. Soc. Sci. 28, 39–49. doi: 10.1016/j.erss.2017.04.001

[ref12] Chabay I. Koch L. Martinez G. Scholz G. (2019). Influence of narratives of vision and identity on collective behavior change. Sustainability 11:5680. doi: 10.3390/su11205680

[ref13] Dales A. Padfield R. Bridge G. (2024). Critical sustainability stories: how communicators can translate the complexity of the climate emergency and shape public narratives. Geoforum 150:103963. doi: 10.1016/j.geoforum.2024.103963

[ref14] De Meyer K. Coren E. McCaffrey M. Slean C. (2021). Transforming the stories we tell about climate change: from 'issue' to 'action'. Environ. Res. Lett. 16:015002. doi: 10.1088/1748-9326/abcd5a

[ref15] Feldman L. Hart P. S. (2021). Upping the ante? The effects of “emergency” and “crisis” framing in climate change news. Clim. Chang. 169:10. doi: 10.1007/s10584-021-03219-5

[ref16] Ferranti E. J. S. Wong J. H. Y. Dhesi S. (2021). A comparison of government communication of climate change in Hong Kong and United Kingdom. Weather Clim. Soc. 13, 287–302. doi: 10.1175/wcas-d-20-0123.1

[ref17] Fisher W. R. (1984). Narration as a human communication paradigm: the case of public moral argument. Commun. Monogr. 51, 1–22. doi: 10.1080/03637758409390180

[ref18] Fitzgerald J. Wolak J. (2016). The roots of trust in local government in western Europe. Int. Polit. Sci. Rev. 37, 130–146. doi: 10.1177/0192512114545119

[ref19] Fonseca A. Castro P. (2026). Narratives of youth climate activism: exploring the diversity of meaning-making on climate change and citizenship. J. Environ. Psychol. 112:Article 103044. doi: 10.1016/j.jenvp.2026.103044

[ref21] Hajer M. A. (1995). The Politics of Environmental Discourse: Ecological Modernization and the Policy Process. Oxford, UK: Clarendon Press.

[ref22] Han M. S. Zhang Y. W. Liu C. N. (2025). How do two facets of social media interaction shape waste sorting behaviour? Empirical evidence from Beijing, China. Technol. Forecast. Soc. Change 210:123850. doi: 10.1016/j.techfore.2024.123850

[ref23] Hand M. C. Morris M. Rai V. (2023). The role of policy narrators during crisis: a micro-level analysis of the sourcing, synthesizing, and sharing of policy narratives in rural Texas. Policy Stud. J. 51, 843–868. doi: 10.1111/psj.12501

[ref24] Hardy B. (1968). Towards a poetics of fiction: 3) An approach through narrative. NOVEL: A Forum on Fiction 2, 5–14. doi: 10.2307/1344792

[ref25] Heeren A. Mouguiama-Daouda C. Contreras A. (2022). On climate anxiety and the threat it may pose to daily life functioning and adaptation: a study among European and African French-speaking participants. Clim. Chang. 173:15. doi: 10.1007/s10584-022-03402-2, 35912274 PMC9326410

[ref26] Hong Y. Chen T. Lee Y. Jun C. Chen J. Kim J.-S. (2025). Knowledge and acceptance of drought policies: the mediating effects of trust in government and perceived efficacy. J. Environ. Manag. 378:124785. doi: 10.1016/j.jenvman.2025.124785 40043565

[ref27] Hornsey M. J. Fielding K. S. (2016). A cautionary note about messages of hope: focusing on progress in reducing carbon emissions weakens mitigation motivation. Glob. Environ. Change 39, 26–34. doi: 10.1016/j.gloenvcha.2016.04.003

[ref28] Huang J. L. Guo H. Y. (2024). When a bleak future comes closer: interaction effects of emotion and temporal distance framing in climate change communication. BMC Psychol. 12:677. doi: 10.1186/s40359-024-02183-w, 39563456 PMC11577877

[ref29] Hulme M. (2007). Understanding climate change – the power and the limit of science. Weather 62, 243–244. doi: 10.1002/wea.108

[ref30] Husain-Naviatti A. (2025). The power of human narrative: inspiring action on climate change. Environ. Sci. Pol. 163:103954. doi: 10.1016/j.envsci.2024.103954

[ref31] Innocenti M. Santarelli G. Lombardi G. S. Ciabini L. Zjalic D. Di Russo M. . (2023). How can climate change anxiety induce both pro-environmental behaviours and eco-paralysis? The mediating role of general self-efficacy. Int. J. Environ. Res. Public Health 20:3085. doi: 10.3390/ijerph20043085, 36833780 PMC9960236

[ref32] Jones M. D. McBeth M. K. (2010). A narrative policy framework: clear enough to be wrong? Policy Stud. J. 38, 329–353. doi: 10.1111/j.1541-0072.2010.00364.x

[ref33] Jugert P. Greenaway K. H. Barth M. Büchner R. Eisentraut S. Fritsche I. (2016). Collective efficacy increases pro-environmental intentions through increasing self-efficacy. J. Environ. Psychol. 48, 12–23. doi: 10.1016/j.jenvp.2016.08.003

[ref34] Kim S. W. Doh S. (2022). A study on effects of Trust in Government on Trust in Fire Response Depending on self-efficacy [정부 신뢰가 자기효능감에 따라 화재 대응 신뢰에 미치는 영향]. Cult. Conver. 44, 431–441. doi: 10.33645/cnc.2022.7.44.7.431

[ref35] László J. (2008). The Science of Stories: An Introduction to Narrative Psychology. London, UK: Routledge.

[ref36] Lauren N. Fielding K. S. Smith L. Louis W. R. (2016). You did, so you can and you will: self-efficacy as a mediator of spillover from easy to more difficult pro-environmental behaviour. J. Environ. Psychol. 48, 191–199. doi: 10.1016/j.jenvp.2016.10.004

[ref37] Li L. J. (2016). Reassessing trust in the central government: evidence from five national surveys. China Q. 225, 100–121. doi: 10.1017/s0305741015001629

[ref38] Lohkamp M. Farjam M. Brüggemann M. (2026). Assessing threat and efficacy: ideological influences in climate change reporting in US and German newspapers (2006-2023). Environ. Commun. 20, 1060–1074. doi: 10.1080/17524032.2026.2616456

[ref39] Mai L. von Sikorski C. (2025). Narratives are key: how narratives influence solutions journalism and promote climate-friendly behavior. Media Psychol., 1–26. doi: 10.1080/15213269.2025.2567363

[ref40] Manzo K. (2010). Beyond polar bears? Re-envisioning climate change. Meteorol. Appl. 17, 196–208. doi: 10.1002/met.193

[ref41] Merkel S. H. Person A. M. Peppler R. A. Melcher S. M. (2020). Climate change communication: examining the social and cognitive barriers to productive environmental communication. Soc. Sci. Q. 101, 2085–2100. doi: 10.1111/ssqu.12843

[ref9001] Mertova P. Webster L. (2019). Using Narrative Inquiry as a Research Method: An Introduction to Critical Event Narrative Analysis in Research, Teaching and Professional Practice. 2nd ed. London: Routledge. doi: 10.4324/9780429424533

[ref42] Metag J. Schäfer M. Füchslin T. Barsuhn T. Kleinen-von Königslöw K. (2016). Perceptions of climate change imagery: evoked salience and self-efficacy in Germany, Switzerland, and Austria. Sci. Commun. 38, 197–227. doi: 10.1177/1075547016635181

[ref43] Metzger M. J. Flanagin A. J. (2013). Credibility and trust of information in online environments: the use of cognitive heuristics. J. Pragmat. 59, 210–220. doi: 10.1016/j.pragma.2013.07.012

[ref44] Milfont T. L. Sibley C. G. (2012). The big five personality traits and environmental engagement: associations at the individual and societal level. J. Environ. Psychol. 32, 187–195. doi: 10.1016/j.jenvp.2011.12.006

[ref45] Moezzi M. Janda K. B. Rotmann S. (2017). Using stories, narratives, and storytelling in energy and climate change research. Energy Res. Soc. Sci. 31, 1–10. doi: 10.1016/j.erss.2017.06.034

[ref46] Moser S. C. (2010). Communicating climate change: history, challenges, process and future directions. Wiley Interdiscip. Rev. Clim. Chang. 1, 31–53. doi: 10.1002/wcc.11

[ref47] Muluneh M. G. (2021). Impact of climate change on biodiversity and food security: a global perspective—a review article. Agric. Food Secur. 10:Article 36. doi: 10.1186/s40066-021-00318-5

[ref48] Nadeau C. Milkoreit M. Eriksen T. H. Hessen D. O. (2024). Missing the (tipping) point: the effect of information about climate tipping points on public risk perceptions in Norway. Earth Syst. Dynam. 15, 969–985. doi: 10.5194/esd-15-969-2024

[ref49] Neef N. E. Fusswinkel S. Roos C. Frank L. Shihepo K. Richter I. (2023). Optimistic narrative future visions: a communication tool for promoting sustainable (plastic) behavior. Front. Psychol. 14:1252895. doi: 10.3389/fpsyg.2023.1252895, 37790233 PMC10543889

[ref50] O'Neill S. Nicholson-Cole S. (2009). “Fear won't do it” promoting positive engagement with climate change through visual and iconic representations. Sci. Commun. 30, 355–379. doi: 10.1177/1075547008329201

[ref51] Overgaard C. S. B. (2023). Mitigating the consequences of negative news: how constructive journalism enhances self-efficacy and news credibility. Journalism 24, 1424–1441. doi: 10.1177/14648849211062738

[ref52] Pihkala P. (2020). Eco-anxiety and environmental education. Sustainability 12:10149. doi: 10.3390/su122310149

[ref53] Porcuna-Ferrer A. Calvet-Mir L. Faye N. F. Klappoth B. Reyes-Garcia V. Labeyrie V. (2024). Drought-tolerant indigenous crops decline in the face of climate change: a political agroecology account from South-Eastern Senegal. J. Rural. Stud. 105:103163. doi: 10.1016/j.jrurstud.2023.103163, 42574925

[ref54] Randall R. (2009). Loss and climate change: the cost of parallel narratives. Ecopsychology 1, 118–129. doi: 10.1089/eco.2009.0034

[ref55] Reser J. P. Bradley G. L. Ellul M. C. (2014). Encountering climate change: 'seeing' is more than 'believing'. Wiley Interdiscip. Rev. Clim. Chang. 5, 521–537. doi: 10.1002/wcc.286

[ref56] Said N. Wölfl V. (2025). Impact of constructive narratives about climate change on learned helplessness and motivation to engage in climate action. Environ. Behav. 57, 75–117. doi: 10.1177/00139165251315576

[ref57] Shanahan E. A. Deleo R. A. Anderson D. Taylor K. Birkland T. A. Chow C. M. . (2026). How proximity and trust of policy narrators motivate their audience. J. Public Policy 46, 170–186. doi: 10.1017/s0143814x25100937

[ref58] Shanahan E. A. McBeth M. K. Hathaway P. L. (2011). Narrative policy framework: the influence of media policy narratives on public opinion. Policy Polit. 39, 373–400. doi: 10.1111/j.1747-1346.2011.00295.x

[ref59] Smith N. Leiserowitz A. (2014). The role of emotion in global warming policy support and opposition. Risk Anal. 34, 937–948. doi: 10.1111/risa.12140, 24219420 PMC4298023

[ref60] Stecula D. A. Merkley E. (2019). Framing climate change: economics, ideology, and uncertainty in American news media content from 1988 to 2014. Front. Commun. 4:Article 6. doi: 10.3389/fcomm.2019.00006

[ref61] Stern P. C. (2000). Toward a coherent theory of environmentally significant behavior. J. Soc. Issues 56, 407–424. doi: 10.1111/0022-4537.00175

[ref62] Tabernero C. Hernández B. (2011). Self-efficacy and intrinsic motivation guiding environmental behavior. Environ. Behav. 43, 658–675. doi: 10.1177/0013916510379759, 42475469

[ref63] Tai K.-T. Awasthi P. Lee I. P. (2024). Open government data and self-efficacy: the empirical evidence of micro foundation via survey experiments. Gov. Inf. Q. 41:101975. doi: 10.1016/j.giq.2024.101975

[ref64] Thier K. Lin T. (2022). How solutions journalism shapes support for collective climate change adaptation. Environ. Commun. 16, 1027–1045. doi: 10.1080/17524032.2022.2143842

[ref65] Thier K. Wu X. M. (2025). Framing climate solutions: an exploratory quantitative content analysis. Environ. Commun. 19, 359–375. doi: 10.1080/17524032.2024.2396978

[ref66] Troy C. L. C. (2025). Equipping audiences without silver bullets: goals and challenges of climate solutions journalism. J. Pract., 1–20. doi: 10.1080/17512786.2025.2496943

[ref67] Van Valkengoed A. M. Perlaviciute G. Steg L. (2024). From believing in climate change to adapting to climate change: the role of risk perception and efficacy beliefs. Risk Anal. 44, 553–565. doi: 10.1111/risa.14193, 37468444

[ref68] Veland S. Scoville-Simonds M. Gram-Hanssen I. Schorre A. K. El Khoury A. Nordbo M. J. . (2018). Narrative matters for sustainability: the transformative role of storytelling in realizing 1.5°C futures. Curr. Opin. Environ. Sustain. 31, 41–47. doi: 10.1016/j.cosust.2017.12.005

[ref70] Yan Z. X. Arpan L. Raney A. (2026). Eliciting empathy and anticipated guilt to promote pro-environmental actions: the impact of narrative and psychological distance in stories about climate-change impacts on animals. Humanit. Soc. Sci. Commun. 13:13, Article 633. doi: 10.1057/s41599-026-06938-1

[ref71] Yoong S. W. Bojei J. Osman S. Hashim N. H. (2018). Perceived self-efficacy and its role in fostering pro-environmental attitude and behaviours. Asian J. Bus. Account. 11, 151–186. doi: 10.22452/ajba.vol11no2.5

[ref72] Zhan S. Wang Y. Yang Y. (2025). How internet use affects hierarchical political trust pattern?—empirical analysis based on CSS2021 data. Public Adm. Policy Rev. 14, 87–100. doi: 10.3969/j.issn.2095-4026.2025.02.007

[ref9002] Zhang Y. Orbie J. (2021). Strategic narratives in China’s climate policy: analysing three phases in China’s discourse coalition. Pac. Rev. 34, 1–28. doi: 10.1080/09512748.2019.1637366

[ref73] Zhou L. (2007). Governing China’s local officials: an analysis of promotion tournament model. Econ. Res. J. 42, 36–50.

