# Supplementary material for: Problem-oriented or solution-oriented? The effects of climate narratives on pro-environmental behavioral intention
Source: Front Psychol. 2026 Aug 24;17:1878805. doi: 10.3389/fpsyg.2026.1878805 (PMC13547036; doi:10.3389/fpsyg.2026.1878805)
Supplement: Supplementary file 1 [file Supplementary_file_1.DOCX]

**Appendix: Manipulation Materials**

**A1: Problems-oriented Climate Narrative**

**English Version:**

The global climate system is warming at an unprecedented rate. In 2025, the global mean surface temperature rose 1.40°C above pre-industrial levels (1850–1900) and 0.52°C above the long-term average, making it one of the warmest years on meteorological record. The Sixth Assessment Report of the Intergovernmental Panel on Climate Change (IPCC) confirms that climate change is primarily driven by human activities, with rising greenhouse gas concentrations being the principal cause of global warming.

If greenhouse gas emissions continue to increase, global warming may approach critical climate tipping points. According to the World Meteorological Organization, the frequency of extreme heat events has increased by nearly 50% over the past two decades, while climate-related natural disasters have tripled compared to the 1980s. In 2024 alone, economic losses from extreme weather events exceeded $300 billion globally. The impacts of climate change extend far beyond the natural environment, cascading through water scarcity, crop failures, and infrastructure damage into agricultural production, urban operations, energy supply, public health, and social stability—creating profound uncertainties for human survival and development.

Climate change is no longer a distant future threat but a present reality. The scientific community and climate experts widely recognize that global warming has become an urgent challenge requiring collective societal action. Without proactive intervention, extreme weather events and resource pressures will continue to accumulate, imposing greater uncertainty and tangible risks on future urban security, agricultural stability, and public well-being.

**Chinese Version (used in the study):**

全球气候系统正以前所未有的速度持续变暖。2025年，全球地表平均温度较工业化前（1850—1900年）水平升高1.40℃，较常年值偏高0.52℃，成为有气象记录以来最暖的年份之一。联合国政府间气候变化专门委员会（IPCC）第六次评估报告指出，气候变化主要由人类活动造成，温室气体浓度持续升高是引起全球变暖的主要原因。

如果温室气体排放继续增长，全球升温可能逼近气候临界点。据世界气象组织数据，过去20年间，全球极端高温事件的发生频率增加了近50%，与气候相关的自然灾害数量较1980年代增长了约两倍。仅2024年，全球因极端天气造成的经济损失就超过3000亿美元。气候变化带来的影响不会仅停留在自然环境层面，还将通过水资源短缺、粮食减产、基础设施受损等途径，进一步传导至农业生产、城市运行、能源供应、公共健康和社会安全等领域，使人类生存与发展面临更为严峻的不确定性。

气候变化已不再是遥远的未来威胁，而是正在发生的现实。科学界与气候专家普遍认为，气候变暖已成为全社会需要共同面对的紧迫挑战。若不积极采取行动，极端天气与资源压力将持续累积，给未来城市安全、农业稳定和公众生活带来更大的不确定性与现实压力。

**A2: Solutions-oriented Climate Narrative**

**English Version:**

The global climate system is warming at an unprecedented rate. In 2025, the global mean surface temperature rose 1.40°C above pre-industrial levels (1850–1900) and 0.52°C above the long-term average, making it one of the warmest years on meteorological record. The Sixth Assessment Report of the Intergovernmental Panel on Climate Change (IPCC) confirms that climate change is primarily driven by human activities, with rising greenhouse gas concentrations being the principal cause of global warming.

Addressing climate change has become an irreversible global trend. In terms of climate adaptation, 171 countries have now established at least one national-level adaptation policy, strategy, or plan, with an increasing number integrating adaptation actions as key components of their Nationally Determined Contributions (NDCs). Regarding energy transition, countries are accelerating the replacement of fossil fuels with renewable energy, promoting large-scale deployment of wind and solar power, and employing policy instruments such as carbon pricing, green subsidies, and energy efficiency standards to guide societal emissions reductions. At the individual level, research demonstrates that an average household can reduce approximately 2 tons of carbon emissions annually through behavioral adjustments such as choosing public transportation, minimizing food waste, and optimizing indoor temperature control.

Climate change need not be passively endured—it can be actively addressed through collective action. The scientific community and climate experts widely recognize that climate governance requires whole-of-society participation. Only when every individual becomes an active participant in climate action can we better safeguard urban security, maintain agricultural stability, enhance quality of life, and secure a more sustainable future for our planet.

**Chinese Version (used in the study):**

全球气候系统正以前所未有的速度持续变暖。2025年，全球地表平均温度较工业化前（1850—1900年）水平升高1.40℃，较常年值偏高0.52℃，成为有气象记录以来最暖的年份之一。联合国政府间气候变化专门委员会（IPCC）第六次评估报告指出，气候变化主要由人类活动造成，温室气体浓度持续升高是引起全球变暖的主要原因。

应对气候变化已成为不可阻挡的全球趋势。在适应气候变化方面，目前已有171个国家制定至少一项国家级适应政策、战略或计划，越来越多的国家将适应行动纳入国家自主贡献的关键组成部分。在能源转型方面，各国正加快以可再生能源替代化石能源，推动风电、光伏等清洁能源的大规模部署，并通过碳定价、绿色补贴和能效标准等政策工具引导社会减排。在日常生活层面，研究表明一个普通家庭通过选择公共交通、减少食物浪费、合理调节室内温度等行为调整，每年可减少约2吨碳排放。

气候变化并非只能被动承受，而是可以通过行动加以应对。科学界与气候专家普遍认为，气候治理要求全社会共同参与。只有人人都成为气候行动的积极参与者，才能更好地保障城市安全、维护农业稳定、提升生活品质，为地球赢得更加可持续的未来。
